# A Prospective Observational Comparison of Two Approaches to Anesthetizing the Trachea for Awake Intubation

**DOI:** 10.7759/cureus.22440

**Published:** 2022-02-21

**Authors:** Thomas M McCutchen, Kathleen N Johnson, Jacob G Fowler, Jessica E Fanelli, Saskia C Anzola, Sarah J Bost, Thomas W Templeton, Amit K Saha

**Affiliations:** 1 Anesthesiology, Wake Forest School of Medicine, Winston-Salem, USA; 2 Anesthesiology, George Washington School of Medicine, Washington, DC, USA; 3 Anesthesiology, Tulane University School of Medicine, New Orleans, USA

**Keywords:** fiberoptic intubation, trans-tracheal block, tracheal anesthesia, awake intubation, airway management

## Abstract

Background: Multiple techniques have been described for anesthetizing the lower glottis and trachea prior to awake fiberoptic intubation. The primary aim of this study is to evaluate whether direct application of local anesthetic to the lower airway via an epidural catheter under direct vision is equally efficacious when compared to use of a transtracheal block in adult patients with an anticipated difficult airway.

Methods: Patients age >18 years requiring awake fiberoptic intubation who underwent upper and lower airway topicalization were observed prospectively. Following topicalization of the upper airway, patients underwent either a transtracheal block or had their trachea and lower glottis anesthetized under direct vision via dispersion of local anesthetic through a multi-orifice epidural catheter. Choice of technique was at the discretion of the attending anesthesiologist. The primary outcome was defined as the degree of coughing observed at the time of intubation based on a 4-point ordinal scale.

Results: Awake intubations in 88 patients were observed with 44 patients undergoing transtracheal block and 44 patients undergoing the epidural catheter technique. Degree of coughing with intubation was similar for each approach with a coughing score of (0, IQR (0,1)) versus (0, IQR (0,1)) in the epidural catheter and transtracheal groups respectively (p = 0.385). Duration of procedure was less in the transtracheal group (1.35 ± 1.54 min) vs. epidural catheter approach (2.86 ± 2.20 min) (p< 0.001).

Conclusion: The epidural catheter and transtracheal approach appear to be equally effective at preventing coughing with intubation during awake fiberoptic intubation.

## Introduction

Awake fiberoptic intubation remains the gold standard for securing the airway in situations where the clinician is concerned that mask ventilation and intubation will be challenging. Successful awake intubations require proper sedation and topicalization of the patient's airway. Coughing and gagging during attempts to secure the airway make the intubation more difficult for the clinician and more unpleasant for the patient.

Crucially, the trachea and lower glottis must be anesthetized to ablate the cough reflex during awake intubation. This can be accomplished using a transtracheal block (TTB) or by topicalizing these structures under direct vision using a multi-orifice epidural catheter that has been passed through the suction port of a fiberoptic scope [1-6]. The epidural catheter technique (ECT) for anesthetizing the airway distal to the larynx in awake fiberoptic intubation has been described in multiple case studies and shows promise as an alternative to the standard TTB when the former is absolutely or relatively contraindicated [6-8]. Contraindications may include distorted anatomy due to neck masses, infection, postsurgical or radiation changes, or trauma making the subglottic trachea difficult or impossible to locate with a needle. There remains, however, a lack of literature establishing the efficacy of this approach in comparison to a TTB.

The primary aim of this prospective observational study is to assess the efficacy of anesthetizing the lower airway via direct vision and dispersion of local anesthetic using a multi-orifice epidural catheter compared to using a TTB. Further, we hypothesize that the ECT may be equivalent to the TTB in ablating the cough reflex.

## Materials and methods

After IRB approval (Protocol #3728, Wake Forest University Health Sciences, Winston-Salem, NC, USA) we performed a prospective, observational study examining two different approaches to anesthetizing the lower airway in preparation for awake intubations. A waiver of consent was granted as there were no specific study interventions. The study was approved by our local Institutional Review Board as a strictly observational study because both interventions represent current standards of care at our institution, patients were not randomized to a specific intervention, and they required awake intubation because of the need for surgery in the presence of a difficult airway, cervical spinal cord compression, cervical spine instability, or some combination of the three. The appropriate EQUATOR checklist was used in the preparation of this manuscript. 

Inclusion criteria included the need for awake intubation with a flexible fiberoptic bronchoscope as judged by the attending anesthesiologist in which anticipated tracheal anesthesia was to be performed using either transtracheal block or the epidural catheter technique. Indications for awake fiberoptic intubation fell into two categories. The first was concern for the potential for a can’t intubate/can’t ventilate scenario if intubation were to be attempted under general anesthesia. Concern was prompted by history of a known difficult airway and/or physical characteristics concerning for a potentially difficult airway. The second was perceived threat to the cervical spinal cord with intubation under general anesthesia because of cervical spinal cord compression, cervical spine instability, or both. Patients in the study underwent a wide variety of surgical procedures, predominately cervical spine procedures or head and neck surgery. The decision of whether to use TTB versus the ECT was solely at the discretion of the attending anesthesiologist caring for the patient and was not dictated by the study or study personnel. Patients were not randomized to either technique. Patients under the age of 18 were not observed. Patients in which tracheal anesthesia was accomplished by a technique other than TTB or ECT were not observed or included in the study.

Demographic information including age, weight, height, body mass index (BMI), gender, and American Society of Anesthesiologists (ASA) status was collected. The airway exam including Mallampati classification, thyromental distance, and oral aperture was recorded along with the patient’s neck circumference, procedure, indication for the awake intubation, and major comorbidities. Additionally, we recorded the level of training of the person anesthetizing the lower airway and performing the intubation.

The prescription for sedation during airway topicalization was determined by the anesthesiologist performing the case based on his or her individual preferences. Typically, sedation was administered in both the holding room, as well as in the operating room prior to beginning airway topicalization. Additional sedation was administered during the intubation at the discretion of the anesthesiologist. Approaches to anesthetizing the upper airway included using a 4% lidocaine nebulizer treatment, 5% lidocaine paste applied to mucosal surfaces, 4% lidocaine atomizer sprays to mucosal surfaces, and landmark-based glossopharyngeal nerve and superior laryngeal nerve blocks.

Following topicalization of the upper airway, one of two different approaches was used to anesthetize the lower airway based on the preference of the anesthesiologist. In patients in whom a TTB was used the clinician initially identified the cricothyroid membrane via anatomic landmarks (ultrasound was not used to identify the cricothyroid membrane in any of the observed patients). Following skin preparation with isopropyl alcohol, the clinician inserted a 21G needle through the cricothyroid membrane. Once in the trachea, air was aspirated to confirm placement and 4 mL of 4% lidocaine was administered. The airway was then secured in standard fashion with an endotracheal tube being railroaded off a flexible fiberoptic scope once the distal tip of the flexible fiberoptic scope was present within the trachea.

**Figure 1:**
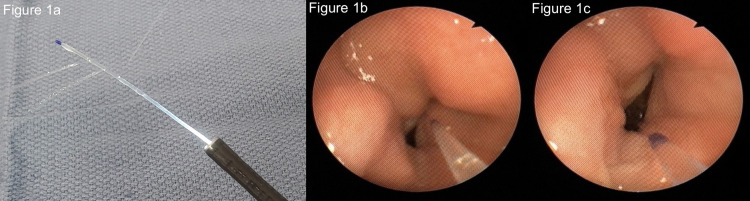
The pictures above illustrate the epidural catheter technique for topicalizing the subglottic trachea. A multi-orifice epidural catheter is passed through the suction port of a flexible fiberoptic bronchoscope (A). Prior to pharyngoscopy, its tip is positioned just proximal to the end of the scope. The nasopharynx or oropharynx, hypopharynx, and larynx are anesthetized. The fiberoptic scope is then positioned just proximal to the larynx and the epidural catheter is advanced into the trachea under direct visualization (B and C). Local anesthetic is injected through the catheter to anesthetize the tracheal mucosa. The scope is then withdrawn or left in position just proximal to the larynx and an appropriate amount of time is waited for the local anesthetic to numb the tracheal mucosa. The patient is then intubated.

In patients in which the lower airway was anesthetized under direct vision, a 21G multi-orifice epidural catheter was inserted through the instrument channel of a flexible fiberoptic bronchoscope (Figure 1). To save costs, individually packaged 21G multi-orifice epidural catheters were used (Portex, Smiths Medical, Keene, NH, USA). After topicalization of the upper airway, the fiberoptic scope was navigated trans-orally then positioned just proximal to the larynx and the epidural catheter was advanced into the trachea under direct visualization (Figures 1B and 1C). Four mL of 4% lidocaine were injected through the catheter to anesthetize the lower glottis and tracheal mucosa. The scope was then withdrawn or left in position just proximal to the larynx. The patient was then intubated with the flexible fiberoptic bronchoscope in typical fashion by railroading the endotracheal tube off of the fiberoptic scope into the trachea once the distal end of the fiberoptic scope was present within the trachea.

The primary outcome of the study was defined as the incidence and severity of coughing during intubation and was assessed by independent observers not involved in the care of the patient using the following scale: 0 - no coughing or gagging, 1 - mild coughing with advancement of the endotracheal tube into the trachea, 2 - moderate coughing with intratracheal placement of the endotracheal tube, 3 - severe coughing and/or gagging that made intubation difficult, 4 - severe coughing and/or gagging that required additional local anesthetic and/or change in technique to achieve successful intubation. The independent observers were clinical research technicians familiar with the study design employed by the Department of Anesthesiology at the Wake Forest University School of Medicine. Observers were not blinded to the technique used to anesthetize the trachea.

Secondary outcomes included time from beginning lower airway topicalization to successful intubation. This was measured using a stopwatch by the clinical research technicians. Ease of lower airway block placement was also assessed using the following scale: 1 - one pass with the needle in the case of the TTB or no repositioning required and one attempt at correctly advancing the epidural catheter into the trachea, 2 - multiple repositioning attempts or multiple needle redirects required, 3 - two or more operators required, 4 - technique abandoned. Additionally, ease of placement was rated by the clinician performing the lower airway technique on a scale of 1-10, where 1 represents the greatest ease and 10 is technically impossible. Other details collected included type and amount of sedation medications administered, and approach and medications used for upper airway topicalization.

Statistical analysis

Given the anticipated low sample size for evaluating non-inferiority in the primary outcome of coughing based on a 4-point ordinal scale and its inability to potentially evaluate secondary outcomes, we developed our sample size based on the secondary outcome of time to intubate following initiation of block placement. An initial query at the time of project development indicated the mean time spent in performing the lower airway topicalization technique with TTB is 1.5 min with a standard deviation of 1.6 min. The sample size of 44 per group was determined to detect a one-minute difference in time spent performing the lower airway topicalization and intubation between the two treatment arms with a two-sided type I error of 0.05 and power of 80%.

Descriptive statistics were performed on all demographic data. Normally distributed data are presented as means with standard deviation, and data that were not normally distributed are presented as medians with inter-quartile ranges. Categorical covariates were compared using the chi square test for proportions. Continuous covariates were evaluated for normality using a Shapiro-Wilk Normality test and Kolmogorov-Smirnov goodness-of-fit test.

The primary outcome of coughing was evaluated using a Mann-Whitney U test for comparison of non-parametric data. The secondary outcome of time from beginning to anesthetize the lower airway to successful intubation was first evaluated for normality using a Shapiro-Wilk Normality test and Kolmogorov-Smirnov goodness-of-fit test. These were then compared using an independent, two-tailed t-test. Other ordinal and non-continuous outcomes were evaluated using a Mann-Whitney U test for comparison of non-parametric data. A p-value of < 0.05 was considered statistically significant. Data were analyzed using Statistical Package for Social Sciences (SPSS) version 26 (IBM Corp., Armonk, NY, USA).

Data are available on reasonable request. The data are stored as de-identified participant data which are available on request to Thomas M. McCutchen, MD ().

## Results

Demographics 

Between November 8, 2019 and May 14, 2021, 88 patients were evaluated in the study. Demographics for these patients are summarized in Table 1. All patients were intubated orally using a flexible fiberoptic bronchoscope. There were no significant differences between either group.

**Table 1:** Demographics Information for the Study population

Characteristics	Epidural Catheter Technique (n = 44 )	Transtracheal Block (n = 44)	p-Value
Age (yr) (Mean, SD)	63.45, 10.5	59.66, 1.69	0.113
Weight (kg) ( Mean, SD)	101.23. 30.86	94.09, 21.65	0.213
Height (m) (Mean, SD)	1.75, 0.09	1.70, 0.11	0.037
Body mass index (kg/m^2^) (Mean, SD)	32.68, 7.89	32.27, 5.96	0.784
Thyromental depth (fingerbreadths) (Mean, SD)	3.05, 0.48	2.88, 0.55	0.144
Oral aperture (fingerbreadths) (Mean, SD)	3.02, 0.34	2.98, 0.348	0.532
Female gender (n)	16	20	0.516
Neck circumference > 44 cm (n)	26	31	0.364

Primary outcome

**Figure 2:**
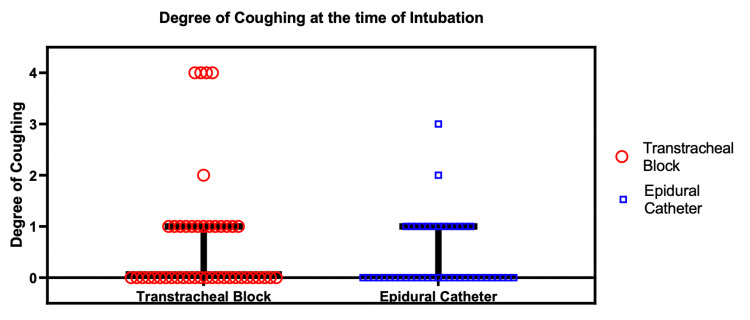
Median and IQR plot for assessment of coughing (4 Scale). Degree of coughing at the time of intubation for transtracheal block (median 0, IRQ (0,1)) and for the epidural catheter (median 0, IRQ(0,1)), p-value = 0.385.

The primary outcome of coughing was equivalent between groups: (0, IQR (0,1)) versus (0, IQR (0,1)) in the ECT and TTB groups, respectively (p = 0.385) (Figure 2). Notably, subgroup analysis for level of training did not show a statistically significant difference in coughing with intubation based on level of experience.

Secondary outcomes

**Figure 3:**
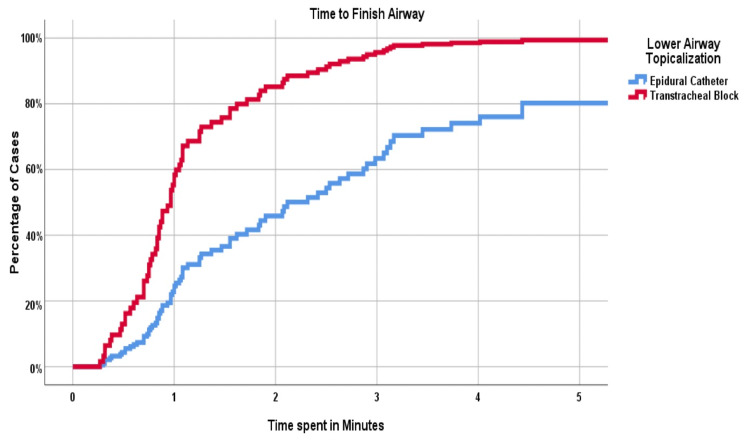
Cox regression model for time to finish airway within 5 minutes.

**Figure 4:**
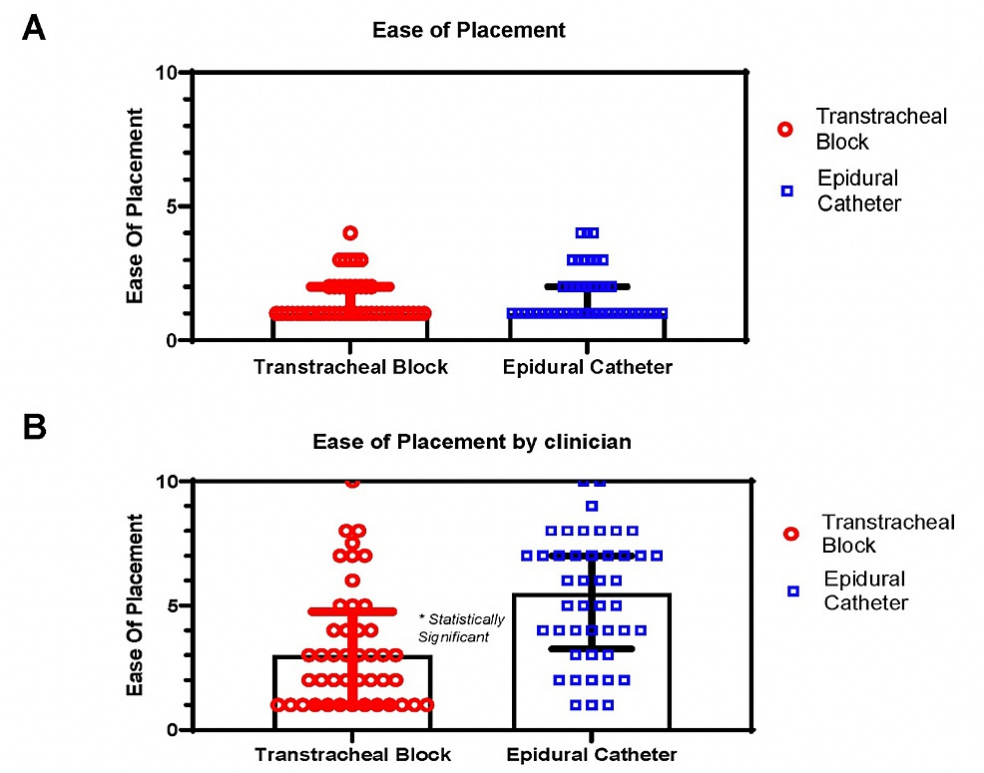
A) Median and IQR Plot for ease of placement (4 Scale). B) Median and IQR plot for ease of placement by clinician (10 Scale). *Statistically significant p < 0.001, **Each point represents a data point

Time from beginning the TTB to securing the airway was faster than using the direct vision and ECT to airway topicalization, 1.35 ± 1.54 min vs. 2.86 ± 2.20 min (p < 0.001). Further this difference persisted after controlling for age, gender, height, neck circumference, Mallampati score, thyromental distance, oral aperture, and ASA classification status (Figure 3 and Table 2). Additionally, we did not observe a difference in the objective assessment of airway block placement (Figure 4A). Interestingly, however, clinicians rated TTB execution to be easier than use of direct vision and an ECT (Figure 4B). 

**Table 2:** Cox regression Model for Time to Finish Airway within 5 minutes

Covariate	Odds ratio	95.0% CI for Odds ratio		p-Value
		Lower	Upper	
Age (yr)	1.016	0.989	1.044	0.237
Weight (kg)	1.034	0.950	1.126	0.433
Height (m)	0.001	0.000	16.569	0.157
BMI (kg/m2)	0.927	0.708	1.215	0.583
Mallampati score of 1 (Ref 4)	0.606	0.252	1.456	0.263
Mallampati score of 2 (Ref 4)	1.567	0.795	3.087	0.194
Mallampati score of 3 (Ref 4)	1.047	0.543	2.018	0.891
Thyromental distance (fingerbreadths)	0.931	0.585	1.482	0.764
Oral aperture (fingerbreadths)	0.611	0.276	1.351	0.223
ASA physical status	0.518	0.259	1.035	0.063
Female gender	1.711	0.411	7.116	0.460
Neck circumference > 44 cm	1.768	0.884	3.534	0.107
Lower airway topicalization (Ref : Epidural Catheter)	0.321	0.182	0.567	< 0.001*

## Discussion

The primary finding of this prospective observational study was that both approaches to anesthetizing the lower airway are effective at preventing coughing with endotracheal tube placement during awake fiberoptic intubation. Additionally, this equivalence persisted across various levels of training. Awake fiberoptic intubation using direct vision in combination with an ECT did take longer to perform; however, the time difference of 90 seconds is likely of little clinical consequence. The ECT approach was perceived as more difficult to execute by clinicians in the study. Interestingly, this finding is consistent even for patients with concerning airway indices and/or large neck circumferences.

Arguably, the ECT may be better tolerated by the patient because it is a needle-free procedure although this advantage is difficult to assess in patients who are sedated and possibly amnestic of the procedure. Perhaps the biggest advantage of the ECT is its ability to provide effective tracheal anesthesia in patients where TTB is absolutely or relatively contraindicated or difficult due to anatomic distortion. In addition, the TTB carries some risk that the ECT does not as it is an invasive procedure. There is a risk of bleeding and hematoma, which could lead to airway obstruction, as well as the possibility of puncturing the posterior tracheal wall and possibly the esophagus [4]. These reasons justify knowledge of and ability to perform the ECT or similar noninvasive techniques prior to awake intubation.

Sethi et al. compared the ECT to the TTB and found that the ECT produced better intubating conditions and less coughing than both the TTB and nebulization [8]. In contrast to this, we found no significant difference in post-intubation coughing in patients who were topicalized with the ECT compared to TTB. Our patients were, however, on average, older and heavier than their patients, which may have resulted in more difficult intubations. In patients with redundant tissue, steering of the FFB may be difficult, which may also make the ECT difficult. Additionally, 12.5% of our patients were smokers, which may have made ablating the gag and cough reflex substantively more difficult. Finally, it is important to note that in other studies comparing these approaches, airway management was performed by the attending anesthesiologist, while our study included airways managed by trainees under the direction of an attending as well as airways managed solely by an attending [6,9].

Transtracheal block may be faster because the ECT is more equipment-intensive and requires more maneuvers to deliver the local anesthetic to the tracheal mucosa. Many facilities will not have access to individually packaged multi-orifice catheters, necessitating opening an entire epidural tray merely to use the catheter whereas a TTB only requires an appropriate needle and syringe. Additionally, successful topicalization of the trachea via the ECT is more reliant on upper airway topicalization (particularly in the distribution of the superior laryngeal nerves) than TTB because the fiberoptic scope must pass through the oropharynx and hypopharynx to allow introduction of the epidural catheter into the trachea. Our study investigated solely spraying local anesthetic directly into the trachea as we focused on lower airway topicalization, rather than supraglottic sprays or the spray-as-you-go technique that other studies have described [8-11]. The spray-as-you-go technique with the epidural catheter may be beneficial to avoid gagging or coughing responses prior to reaching the glottic opening while minimizing or eliminating other maneuvers necessary to topicalize the upper airway. Xue et al. found that patient comfort scores were better during supraglottic and laryngeal sprays with the epidural catheter as compared to the endotracheal sprays. Unfortunately, they also found that the spray-as-you-go ECT took on average 23 minutes to perform which may in some cases be clinically significant [9]. In contrast to this, 50% of the patients in our study were successfully intubated within 2.5 minutes of starting the intratracheal dispersion of local anesthetic via the ECT.

Limitations of this study include the fairly small sample size and single-center nature of the cohort. The inclusion of trainees as well as attending clinicians and their inherent diversity in experience may make our finding less generalizable to other clinical environments in which trainees are not present. Additionally, the lack of a standardized sedation regimen and protocol for upper airway topicalization may have impacted the primary outcome. Point of care ultrasound was not used to identify the cricothyroid membrane, possibly making TTB more difficult, less effective, or more time-consuming to place. Some of the parameters observed (timing the technique chosen to anesthetize the trachea) made blinding of the observers to the chosen technique impossible. Some of the parameters observed (difficulty performing the technique, amount of coughing with intubation) have a degree of inherent subjectivity. Perhaps most significantly, patients were not randomized to receive TTB or ECT; the decision of which technique to use was made by the anesthesiologist caring for the observed patient. Thus the observational only nature of this study may have created a selection bias which may have affected our results. 

## Conclusions

In conclusion, our study found that anesthetizing the lower airway under direct vision using a multi-orifice epidural catheter to disperse local anesthetic was not inferior to TTB in terms of ablating the cough reflex in the setting of awake fiberoptic intubation. Consequently, the ECT provides a good alternative to the TTB in settings where TTB is not a feasible option. Using a transtracheal approach did reduce the time to intubation and therefore, may represent a better choice to anesthetizing the lower airway in settings where urgency and timeliness in securing the airway are of increasing importance.
